# The Oath of Hippocrates

**Published:** 1905-09

**Authors:** 


### THE OATH OF HIPPOCRATES.

WHILE our readers are doubtless familiar with this oath, as usually published in condensed form, the translation in full, furnished by a valued correspondent, and published elsewhere in this number, may be of special interest.

Hippocrates, born on the Island of Cos, about 400 B.C., and dying in Larissa, Thessaly, about 377 B.C., is recognized as the Father of Medicine, probably as much from the high ethical character of this oath as from his writings, voluminous as these were.

The republication of this oath at this time seems appropriate at this season of conventions, for if it have no other object, it will, at least, remind dentists that they are a part of the great healing art, and that the ethical laws, as laid down by the Master, are as forceful in their line of work as in that of general medicine.

There never has been a period when the true professional idea requires inculcation into the dental mind more than to-day. The dental profession is apparently drifting away from the ethical tenets of Hippocrates and absorbing the commercial spirit of the age. The true professional spirit is gradually being lost in the financial sophistries of the hour, practically taught by example in dental colleges, and practised by graduates and practitioners. It is well, therefore, to recall the foundation principles upon which the healing art is founded, and which are as true to-day as they were twenty-four hundred years ago.

